# Managing Select Immune-Related Adverse Events in Patients Treated with Immune Checkpoint Inhibitors

**DOI:** 10.3390/curroncol31100473

**Published:** 2024-10-18

**Authors:** Parneet K. Cheema, Marco A. J. Iafolla, Husam Abdel-Qadir, Andrew B. Bellini, Nazira Chatur, Natasha Chandok, Vikram R. Comondore, Morven Cunningham, Ilana Halperin, Anne B. Hu, Diana Jaskolka, Saeed Darvish-Kazem, Masud H. Khandaker, Abhijat Kitchlu, Jasdip S. Sachdeva, Shane Shapera, Nicholas R. J. Woolnough, Massey Nematollahi

**Affiliations:** 1 William Osler Health System, Brampton, ON L6R 3J7, Canada; marco.iafolla@williamoslerhs.ca (M.A.J.I.); andrew.bellini@williamoslerhs.ca (A.B.B.); natasha.chandok@williamoslerhs.ca (N.C.); vikram.comondore@williamoslerhs.ca (V.R.C.); anne.hu@williamoslerhs.ca (A.B.H.); diana.jaskolka@williamoslerhs.ca (D.J.); saeed.darvish-kazem@williamoslerhs.ca (S.D.-K.); masud.khandaker@williamoslerhs.ca (M.H.K.); jasdip.sachdeva@williamoslerhs.ca (J.S.S.); nicholas.woolnough@williamoslerhs.ca (N.R.J.W.); massey.cns@gmail.com (M.N.); 2 Women’s College Hospital Research Institute, Toronto, ON M5S 1B2, Canada; husam.abdel-qadir@wchospital.ca; 3 Division of Cardiology, Peter Munk Cardiac Centre, Toronto General Hospital, University Health Network, Toronto, ON M5G 2N2, Canada; 4 Division of Gastroenterology, Faculty of Medicine, Vancouver General Hospital (Sanders), University of British Columbia, Vancouver, BC V5Z 1M9, Canada; nazira.chatur@ubc.ca; 5 Toronto Centre for Liver Disease, University Health Network, Toronto, ON M5G 2C4, Canada; morven.cunningham@uhn.ca; 6 Division of Endocrinology, Department of Medicine, Sunnybrook Health Sciences Centre, Toronto, ON M4N 3M5, Canada; ilana.halperin@sunnybrook.ca; 7 Division of Nephrology, Department of Medicine, University Health Network, University of Toronto, Toronto, ON M5G 2C4, Canada; abhijat.kitchlu@uhn.ca; 8 Department of Medicine, University of Toronto, Toronto, ON M5G 2N2, Canada; shane.shapera@uhn.ca

**Keywords:** immune checkpoint inhibitors, immune-related adverse events, immunosuppressants

## Abstract

The increased use of immune checkpoint inhibitors (ICIs) across cancer programs has created the need for standardized monitoring and management of immune-related adverse events (irAEs). Delayed recognition without appropriate treatment can have serious and life-threatening consequences. The management of irAEs presents a unique set of challenges that must be addressed at a multidisciplinary level. Although various national and international guidelines and working groups provide high-level recommendations for the management of irAEs, practical guidance is lacking. Furthermore, timely collaboration between specialists requires institutional protocols that enable the early recognition, assessment, and treatment of irAEs. Such protocols should be developed by institution specialists and include algorithms for all healthcare providers involved in the care of patients treated with ICIs. At William Osler Health System in Brampton, Ontario, practical step-by-step multidisciplinary treatment approaches with recommendations for the management of irAEs were developed in collaboration with experts across Canada. Here, we provide an in-depth description of the approaches, outlining baseline investigations prior to the initiation of ICIs, as well as the monitoring and management of irAEs based on symptoms, severity, and involved organ systems. We encourage other centres to adapt and modify our approaches according to their specific needs and requirements.

## 1. Introduction

Immune checkpoint inhibitors (ICIs) targeting cytotoxic T-lymphocyte-associated antigen-4 (CTLA-4), programmed cell death-1 (PD-1), and PD ligand 1 (PD-L1) have become the standard of care for several cancer types. However, their unique mechanism of action could lead to a spectrum of adverse events (AEs) that are quite different from those associated with traditional cytotoxic chemotherapy [1,2]. Although these immune-related AEs (irAEs) may involve any organ or organ system, the most prevalent are gastrointestinal (GI), dermatologic, hepatic, endocrine, and pulmonary toxicities.

The incidence and onset of irAEs vary based on the class and dose of the ICI administered, the type of cancer, and patient-related factors [3]. In general, patients receiving anti–CTLA-4 agents have a higher incidence of any grade irAEs than those treated with PD-1 or PD-L1 antibodies [4,5,6,7]. The incidence, severity, and onset of irAEs increase when ICIs are used in combinations [8]. All irAEs require early detection and proper treatment as they could lead to life-threatening declines in organ function and quality of life (QoL). Fatal outcomes have been reported [9].

The management of irAEs presents a unique set of challenges that must be addressed at a multidisciplinary level. Several well-established clinical practice guidelines, such as the National Comprehensive Cancer Network (NCCN), the American Society of Clinical Oncology (ASCO), the European Society for Medical Oncology (ESMO), the Society for Immunotherapy of Cancer (SITC), and Cancer Care Ontario (CCO), have provided high-level recommendations on the management of irAEs [10,11,12,13,14]. Yet, thorough practical guidance is lacking. Timely collaboration between specialists requires institutional protocols that enable the early recognition, assessment, and treatment of irAEs in a standardized manner. Such protocols should be developed by multidisciplinary teams and include algorithms to be followed by healthcare providers involved in the care of patients treated with ICIs. Here, we outline practical multidisciplinary approaches to guide the diagnosis and management of common irAEs. These approaches to managing certain irAEs from ICIs were initially developed at William Osler Health System (WOHS) in Brampton, Ontario and then further advanced through collaboration with specialists across Canada including medical oncologists, nephrologists, gastroenterologists, respirologists, cardiologists, hepatologists, and endocrinologists. We describe baseline assessments prior to the ICI initiation, as well as the monitoring and management of irAEs based on symptoms, severity, and involved organ systems. We encourage other centres to adapt and modify our algorithms according to their needs and requirements. Although we provide general guidance on dosing, clinicians should consider the severity of the irAEs and patients’ comorbidities and ability to tolerate standard doses and tailor the doses to individual patients.

## 2. Management of irAEs

### General Principles

**Figure 1:**
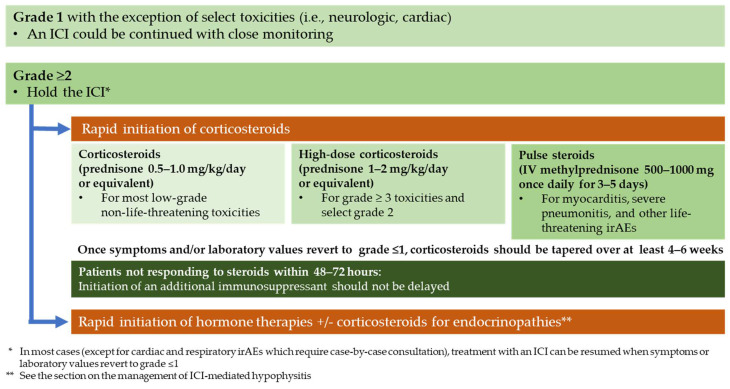
Corticosteroids and immunosuppressants for the management of irAEs: general principles.

Clinical practice guidelines have established several general principles that should be followed when there is a high level of suspicion that the new symptoms are treatment-related, irrespective of the affected organs (Figure 1) [10,11,12,13,14]. For most grade 1 toxicities (except for the presence of some neurologic and cardiac toxicities), the ICI could be continued with close monitoring. It is, however, recommended to perform the following: (1) withhold the ICI for grade ≥ 2 irAEs until the symptoms and/or laboratory values revert to grade ≤ 1; and (2) permanently discontinue the ICI for some grade 3–4 irAEs (e.g., pneumonitis). Additionally, in select cases (i.e., grade 4 cardiac toxicity), experts suggest therapy de-escalation, transitioning, for instance, from combination anti-PD-1–anti-CTLA-4 antibodies to anti-PD-1 monotherapy [15,16].

Corticosteroids are the mainstay first-line therapy for most grade ≥ 2 irAEs [10,11,12,13,14]. Early intervention with steroids is key in achieving rapid irAE resolution. Although an initial dose of prednisone (0.5–1.0 mg/kg/day or equivalent) might be sufficient for grade 2 toxicities, high-dose corticosteroids might be required for patients with neurologic, cardiac, or grade ≥ 3 irAEs. Pulse steroids (IV methylprednisone 500–1000 mg once daily for 3–5 days) could be considered in these scenarios, including in patients with myocarditis, severe pneumonitis, and acute kidney injury (AKI). Once symptoms and/or laboratory values revert to grade ≤ 1, corticosteroids should be tapered over at least 4–6 weeks.

Short-term steroid use has not been shown to reduce the anti-tumour efficacy of ICIs [17]. However, to ensure optimal outcomes and to prevent or mitigate steroid-related AEs and the recurrence of irAEs, necessary prophylactic measures should be undertaken (Table 1). At WOHS, patients with grade ≥ 2 irAEs on a steroid tapering schedule receive proactive calls from a nurse as they taper the steroids.

When patients do not respond to steroids within 48–72 h (steroid-refractory irAEs), the initiation of an additional immunosuppressant, and a consultation with the relevant medical specialist, is warranted. The timely initiation of a second immunosuppressant is crucial [22,23]. Currently, managing steroid-refractory irAEs is primarily based on evidence that is limited to single-centre experiences. Infliximab, mycophenolate mofetil (MMF), tocilizumab, cyclophosphamide, and anti-thymocyte globulin are among the options recommended for managing different steroid-refractory irAEs. However, not all suggested approaches yield positive results for every irAE. For instance, some evidence suggests that infliximab, with or without cyclophosphamide, may not be effective in treating immune-mediated steroid-refractory pneumonitis [24,25]. An ongoing prospective trial (NCT04438382) is currently assessing the efficacy of infliximab combined with intravenous immunoglobulin therapy in managing patients with steroid-refractory pneumonitis. Moreover, there is a preference to avoid using infliximab in patients with myocarditis due to data indicating an increased risk of heart failure [26]. Hence, there is a pressing need for additional approaches. IL-6 receptor blockade using tocilizumab has demonstrated effectiveness in managing AEs stemming from other forms of immunotherapy, particularly cytokine release syndrome (CRS) induced by chimeric antigen receptor (CAR) T-cell therapy [27,28]. Increasing evidence suggests that tocilizumab could also serve as a therapeutic option for addressing steroid-refractory irAEs triggered by immune checkpoint inhibition [29,30,31]. A single-centre experience suggests that, with the exception of infliximab for immune-mediated colitis, tocilizumab could be the treatment of choice for other irAEs in steroid-refractory patients [30]. At WOHS, tocilizumab led to a recovery from immune-related steroid-refractory pneumonitis in 12 out of 15 (80%) patients [31]. The median number of days from the administration of tocilizumab to a documented reduction in supplemental oxygen requirements was 2 (range 0–5). The median number of days from the administration of tocilizumab to a recovery (grade 0 or 1) was 7 (range 1–122). Currently, tocilizumab is listed as an immunosuppressant for steroid-refractory pneumonitis by the SITC guidelines [13] and the ESMO guidelines for steroid-refractory myocarditis, pneumonitis, and hepatitis [12]. However, the evidence supporting tocilizumab’s recommendation in hepatitis is limited and based primarily on case reports [30,31,32].

## 3. Management of ICI-Mediated Colitis

### 3.1. Incidence and Onset

Gastrointestinal (GI) irAEs, such as immune-mediated colitis, are common, occurring in 35–50% of patients receiving ICIs [33]. The events can occur anywhere in the GI tract, including the esophagus, stomach, small bowel, colon, pancreas, gallbladder, bile ducts, and liver, with colitis being the most prevalent [34,35]. The timely diagnosis of GI irAEs is critical because they can escalate rapidly and become life-threatening; symptoms like diarrhea and abdominal pain may progress swiftly to conditions like ileus, toxic megacolon, and bowel perforation [33]. Therefore, clinicians should maintain a high suspicion of immune-mediated colitis when oncology patients treated with ICIs present with diarrhea.

The risk, timing of occurrence, and severity of colitis depend on the type of ICI treatment [36]. Patients treated with CTLA-4 inhibitors have a three times higher risk of ICI-mediated GI events than those treated with PD-1/PD-L1 inhibitors [37,38]. For instance, the overall incidence of diarrhea ranges from 30.2–35.4% for CTLA-4 inhibitors and 12.1–13.7% for PD-1/PD-L1 inhibitors [39,40,41]. Similarly, the incidence of colitis is higher with CTLA-4 inhibitors (5.7–39.1%) compared to PD-1/PD-L1 inhibitors (0.7–31.6%) [39,40,41]. When PD-1/PD-L1 inhibitors and CTLA-4 inhibitors are combined, the reported incidence and severity of colitis tend to be higher. From a retrospective study using the combination of nivolumab and ipilimumab, 32% of patients developed colitis, with 11.2% developing grade ≥ 3 colitis [42]. Complication rates are significantly higher when the ICI combination is given after chemotherapy or with the sequential administration of nivolumab and ipilimumab, in particular with the administration of nivolumab after ipilimumab [43,44].

The onset of ICI-mediated colitis may vary, even occurring after treatment cessation [39], and studies suggest that its onset might vary with ICI type. Studies suggest that colitis induced by CTLA-4 inhibitors tends to appear later than colitis caused by PD-1/PD-L1 inhibitors. For example, the median time to the onset of ipilimumab-induced colitis is around 6–7 weeks, typically after two to three infusions [45,46,47]. As for PD-1/PD-L1 inhibitors, symptomatic colitis is less predictable and could occur as early as 1 week after treatment initiation or up until 2 years after treatment completion [35,48].

Furthermore, the risk of colitis appears to be influenced by the underlying malignancy [49]. Rates of colitis in patients treated with anti-PD-1/PD-L1 inhibitors are higher in patients with melanoma than those with advanced lung cancer or renal cell cancer [39,40]. The exact reasons for the differences are unclear. Table 2 outlines a management algorithm for patients with ICI-mediated colitis.

Infliximab and vedolizumab have different mechanisms of action. While infliximab targets TNF-α to modulate inflammatory responses, vedolizumab acts on integrin receptors to prevent the migration of memory T-lymphocytes into inflamed gastrointestinal tissues. Since there are no direct comparative studies between infliximab and vedolizumab for managing ICI-induced colitis, selecting the appropriate agent depends on various factors, including the patient’s medical history and risk factors. It is important to note that both infliximab and vedolizumab can pose challenges for patients with latent viral or bacterial infections. Although it is standard oncology practice to test for HBV, HIV, and TB prior to the initiation of an ICI, one should ensure that testing is performed prior to the initiation of infliximab or vedolizumab. If there is a history suggestive of prior exposure to HBV or TB, the infliximab administration should be postponed until screening results confirm negativity. Alternatively, vedolizumab could be considered. For patients with a low risk and pending test results, the decision to administer these agents can be made at the discretion of the treating physician; however, it is crucial to not delay treatment if the benefits outweigh the potential risks and pending test results. For patients in whom both infliximab and vedolizumab are contraindicated, other immunosuppressants (i.e., mycophenolate mofetil) could be considered [50].

### 3.2. When to Consider Infliximab or Vedolizumab

According to the SITC guidelines, infliximab or vedolizumab should be considered [13]:

- If diarrhea or colitis symptoms do not respond to corticosteroid therapy within 3–5 days
- If diarrhea or colitis symptoms recur after tapering corticosteroids
- If there is a severe ulcerative presentation on colonoscopy, three doses of infliximab (5 mg/kg) should be administered at 0, 2, and 6 weeks to reduce the risk of colitis recurrence.
- Infliximab is contraindicated in patients with severe infections, such as sepsis, abscesses, tuberculosis, and opportunistic infections, and in patients with moderate or severe (NYHA Class III/IV) congestive heart failure [51].
- Vedolizumab is contraindicated in patients with active severe infections or opportunistic infections but not those with congestive heart failure [52].
- Although current guidelines recommend against infliximab for immune-related hepatitis, this recommendation is based on the observations of infliximab-induced hepatotoxicity in patients with primary autoimmune conditions or IBD who were treated on an ongoing basis and received multiple infliximab treatments over time. There is no published evidence that infliximab induces hepatotoxicity in a dose-limited setting in oncology patients or that infliximab may aggravate steroid-refractory immune-related hepatitis. Contrarily, there are reports of the resolution of immune-related hepatitis with infliximab treatment [53,54].
- Vedolizumab may be considered in patients refractory to infliximab and/or with a contraindication to a TNF-α blocker or in patients with concurrent immune-related colitis and hepatitis.

## 4. Management of ICI-Mediated Hepatitis

### Incidence and Onset

Hepatic toxicity is reported in 1–17% of patients treated with ICIs [8,55]. The incidence varies with the class of drug, and it is higher in patients on dual checkpoint blockade. Hepatitis is most commonly low-grade toxicity; however, grades 3 and 4 have been reported [56].

There is variability in the clinical and histopathologic presentation of ICI-mediated hepatitis, from asymptomatic (the most common initial presentation) or nonspecific symptoms (fever and fatigue) to rapid progression and fulminant disease [57,58]. Clinical features of concern include coagulopathy, development of hepatic encephalopathy (mild confusion or asterixis to coma), ascites, or other symptoms of liver disease (pruritis, jaundice, petechiae) [59]. Since ICIs can lead to multiple inflammatory toxicities, patients with hepatitis and abdominal pain should also be investigated for concurrent GI-related AEs [60]. Although these features are of concern, liver failure due to immune-related hepatitis is rare.

The median onset of ICI-mediated hepatitis is approximately 6–14 weeks after starting the ICI treatment [11,57,61,62,63], and it varies by the underlying malignancy and type of therapy. For example, while the reported median onset of hepatitis in melanoma patients treated with pembrolizumab was 19 weeks (range 3–93), it was 4 weeks (range 1–23 weeks) in those receiving nivolumab. The median onset of hepatitis after nivolumab in lung cancer is 25 weeks (range 4–31) [59,64,65].

It is important to note that ALT elevation can also be found with immune-related myositis/myopathy. Thus, if ALT is elevated, additional testing for AST, GGT (which also tends to be elevated in immune-related hepatitis), CK, and troponin should be considered. B-type natriuretic peptide (BNP) can often help differentiate to determine if there is elevation is due to myositis/myopathy and, if so, if there is a concurrent myocarditis.

The treatment of patients with ICI-mediated hepatitis is complex. It requires a multidisciplinary approach that includes clinicians familiar with the underlying cancer and immunotherapeutic agents, as well as experts in hepatology and liver pathology [66]. Table 3 outlines a treatment algorithm for ICI-mediated hepatitis.

Physicians should also be aware of immune-mediated cholangitis, a rare irAE that can impact both large and small bile ducts. A characteristic biochemical sign of immune-mediated cholangitis is a notable increase in biliary enzymes compared to hepatic enzymes. Unlike immune-mediated hepatitis, glucocorticosteroids appear to be less effective in treating immune-mediated cholangitis. Most immune-mediated cholangitis cases respond partially to immunosuppression. Additional immunosuppressive agents, such as mycophenolate mofetil (MMF), azathioprine, tacrolimus, tocilizumab, and plasmapheresis, have also been used. Ursodeoxycholic acid may improve the liver biochemistry in some patients and is often used, as its toxicity is low. While medical intervention can lead to a decrease in biliary enzyme levels in most patients, achieving normal values may only occur in a minority of cases after 6 to 12 weeks [67].

## 5. Management of ICI-Mediated Nephritis

### Incidence and Onset

Acute kidney injury (AKI) is a known complication in patients on ICI therapy. Its incidence ranges from only 2 to 3% in clinical trials to up to 17% in real-world analyses [18,68,69,70,71,72,73]. Case series of kidney biopsies in patients experiencing ICI-associated AKI report acute interstitial nephritis as the histological diagnosis in over 80% of cases [74,75]. However, various forms of glomerular disease (e.g., podocytopathies, renal vasculitides, and complement-mediated glomerulonephritis) have been reported [74,76]. ICI-mediated nephritis may initially present with increased serum creatinine without any clinical features; however, the patient may progress to anuria, oliguria, edema, and electrolyte disorders [61,77,78]. Renal toxicities often appear after 3 months of treatment but can appear as early as 3 weeks to as late as 30 weeks [61,77,78,79].

The evaluation of ICI-associated nephritis involves a careful determination of its underlying etiology and may require a kidney biopsy to unequivocally establish the diagnosis. In particular, patients presenting with high-grade proteinuria should be considered for kidney biopsy. The management of ICI nephritis requires a multidisciplinary approach to ensure optimal outcomes. Table 4 outlines a management algorithm for ICI-mediated nephritis.

## 6. Management of ICI-Mediated Myocarditis

### Incidence and Onset

Immune-related cardiotoxicity is rare (1–3%) [81,82,83,84] and may include myocarditis, pericarditis, arrhythmias, cardiomyopathy, and left ventricular dysfunction [85,86,87]; however, the high mortality rate historically associated with myocarditis (46%) mandates rapid diagnostic processes and a multidisciplinary approach [1,88,89]. Clinical presentation generally ranges from the asymptomatic elevation of cardiac enzymes to sudden death from heart failure [90].

The first three months of ICI treatment is considered a more likely period for the development of ICI-mediated myocarditis [90,91,92], with a median onset time of 34–65 days [88,89,90,91,92,93]. Although less likely after 3 months of treatment, the occurrence of myocarditis is still possible [89].

Suspected myocarditis is categorized into three groups: definite, probable, and possible [94]. The severity is classified as asymptomatic, non-fulminant, or fulminant. Myocarditis can be ruled in through a combination of symptoms, new increases in troponin, changes in an ECG or echocardiography, and specific MRI features. Biopsy alone is diagnostic but is a last step. While all patients require an ECG, echocardiography, and cardiac troponin assays, assessments may also include ambulatory ECG monitoring (Holter monitoring), cardiac MRI, creatine kinase, and inflammatory biomarkers. B-type natriuretic peptide (BNP) is not a diagnostic biomarker to rule in or rule out myocarditis, but it can be of value in assessing the severity of heart failure. Other potential causes of myocardial injury (e.g., coronary artery disease) should be excluded [93,95]. Early cardiologist referral and in-patient care are recommended.

Some guidelines recommend that patients initiated on ICIs undergo baseline cardiac assessments (i.e., a clinical history and risk factor assessment, an ECG, cardiac troponin, BNP or NT-proBNP, and an echocardiogram) [13,96]. At WOHS, a baseline ECG is performed for patients receiving combination anti-CTLA4/PD1 therapy. Table 5 outlines the algorithm for ICI-mediated myocarditis.

When assessing for ICI-related myocarditis, one should consider that troponin I is elevated in 94% of cases [94], BNP is elevated in 66% of cases [89], and 29% of ICI-related myocarditis cases present with a normal ECG [89,105]. Furthermore, normal ejection fraction is present in 38–55% of cases and therefore does not rule out ICI-related myocarditis [89,105,106,107].

## 7. Management of ICI-Mediated Hypophysitis

### Incidence and Onset

Although ICI-mediated hypophysitis is a relatively common ICI-mediated endocrinopathy, its diagnosis can be challenging because accompanying symptoms may be subtle, the time of onset may be variable, MRI findings may be minimal, and endocrine abnormalities may vary. Prompt diagnosis is, however, important because the correction of hypophysitis can significantly improve a patient’s quality of life. The late detection of hypophysitis, especially when associated with adrenal insufficiency, can lead to high morbidity and mortality [108].

The overall incidence of hypophysitis is 12% in patients treated with anti-CTLA-4 antibodies and 0.5% in patients treated with PD-1 antibodies [109,110,111,112]. It is more frequent in males, and its incidence rises with age [113]. The median time to onset following the ICI administration is 4 months [114]; however, early and late onsets have been reported [115,116]. The risk of hypophysitis with ipilimumab appears to be dose-dependent, with a higher prevalence in those receiving 10 mg/kg vs. 3 mg/kg [117,118,119].

As the clinical features of ICI-induced hypophysitis are nonspecific, an endocrine evaluation should be obtained before each ICI administration for the first 4–6 months so that hypophysitis can be identified and treated early [120,121,122]. Our recommendations for endocrine evaluations are provided in Table 6. Table 7 outlines the management algorithm for patients with ICI-mediated hypophysitis.

- Thyroid dysfunction caused by ICIs can often be managed effectively, and many patients experience recovery with appropriate treatment. Replacement therapy with levothyroxine for hypothyroidism or anti-thyroid medications for hyperthyroidism may be necessary.
  ○ For secondary hypothyroidism, levothyroxine 1.6 µg/kg/day is the standard in endocrine care, although guidelines recommend 0.5–1.5 µg/kg/day.
  ○ If TSH is low but free T4 is low to normal, or in the case of cardiac disease, consider starting at a lower dose of 50–75 µg daily.
- Recovery from immune-related endocrinopathies affecting the sex hormone axis may occur with appropriate management, but individual responses can vary. Testosterone replacement therapy in males and hormone replacement therapy (estrogen and progesterone) in females may be indicated.
- Recovery from immune-related adrenal insufficiency is the least likely and replacement therapy with glucocorticoids (i.e., hydrocortisone) may be necessary.
  ○ If morning cortisol < 250 or random cortisol < 150 nmol/L: hydrocortisone 20 mg (morning), 10 mg (mid-afternoon)

## 8. Management of ICI-Mediated Pneumonitis

### Incidence and Onset

ICI-mediated pneumonitis is a common irAE [124], with a reported incidence of about 3% to 5% [125,126,127,128,129]. The mechanisms leading to pneumonitis are not fully understood. It is believed that the inflammatory state of the lung and the tumour microenvironment are involved [130].

Delayed treatment can have life-threatening consequences [129,131,132], and ICI-mediated pneumonitis accounts for 35% of PD-1/ PD-L1 inhibitor-related deaths [9,12]. The overall fatality rate of ICI-mediated pneumonitis is 10–17% [133].

Current evidence has identified many potential risk factors for ICI-related pneumonitis [134,135,136,137,138,139,140]. These include the patient’s age, gender, previous lung disease, tumour histology, PD-1 blockade, combination therapy, and prior radiation therapy. A higher incidence of ICI-related pneumonitis was observed in males than females and in squamous cells compared to other histological types [135]. Decreased lung function and increased medical complications might increase risk in older adults [141]. Smoking may also play a role in the development of pneumonitis [142].

ICI-mediated pneumonitis occurs more often and has a faster onset in patients with lung cancer than in other types of cancer [126,143]. Evidence indicates that PD-1 inhibitors account for a higher incidence of all-grade (3.6% vs. 1.3%) and high-grade (1.1% vs. 0.4%) pneumonitis when compared to PD-L1 inhibitors [125]. Furthermore, the incidence of pneumonitis is higher when PD-1/PD-L1 inhibitors are used in combination with CTLA-4 inhibitors or sequential with chemotherapy, radiotherapy, or other immunotherapies than when used as a monotherapy [24,128,129,144,145,146,147,148].

The development of ICI-mediated pneumonitis may be accompanied by radiation and/or infectious pneumonia, leading to the simultaneous occurrence of several types of inflammation in the lungs [149]. Further characterization of the unique clinical and radiographic features of ICI-mediated pneumonitis could assist in its diagnosis [150].

The onset of ICI-mediated pneumonitis can occur as early as hours to days or as late as several months after the first ICI dose; however, severe pneumonitis usually has an onset within the first 100 to 200 days of the ICI therapy [151]. It is important to note that ICI-mediated pneumonitis can occur several months after therapy discontinuation; therefore, continuous vigilance after treatment termination is necessary [152]. The management algorithm adopted at WOHS is provided in Table 8.

## 9. Discussion

The selection of an ICI, its dosing, and the decision to use it as a monotherapy or in combination with other treatments should be tailored to the patient’s age and comorbidities to minimize the risk of irAEs. However, these events are often unpredictable. Due to the heterogeneity of cancer types, patient characteristics, and therapeutic approaches—such as whether ICIs are administered as a monotherapy or in combination, and whether they are used as first-line or subsequent therapies—the occurrence, onset, manifestation, and severity of irAEs can vary significantly. This variability impacts their management and necessitates customized approaches based on each patient’s specific presentation.

Current irAE management strategies are informed by evidence from clinical trials, case reports, systematic reviews, and historical experience with various immunosuppressants in treating other immune-mediated diseases. Because managing irAEs often requires a multidisciplinary approach, there are considerable variations in the immunosuppressants used, their dosing, and the duration of the treatment. While our goal is to describe and provide a practical approach to irAE management, we acknowledge that this management will continue to evolve as new ICIs enter the market and as physicians gain experience with different immunosuppressants. Therefore, sharing experiences and publishing outcomes from various approaches is crucial, especially since irAEs may respond to immunosuppressants differently than other immune-related inflammatory diseases.

## 10. Conclusions

Achieving optimal treatment outcomes with ICIs requires a multidisciplinary approach to promptly and effectively recognize and manage irAEs. Here, we provide detailed information and insights on protocols that were initially developed at WOHS as we endeavored to address some of the gaps and challenges in our centre then further expanded to include input from experts across Canada. We outline an approach on how to follow the guideline recommendations in a practical manner and when and how to consult with and engage relevant medical specialists outside the oncology discipline. This document can be useful for oncologists, nurse practitioners, internists, and hospitalists as a framework on how to diagnose and initiate the management of these select irAEs. Although each patient’s care will need to be individualized, this document can provide a framework for oncologists, nurse practitioners, internists, specialists, and hospitalist on the approaches to diagnosing and managing these select irAEs. We encourage other centres to adapt our algorithms according to their institutional needs and requirements.

## Figures and Tables

**Table 1:** Recommendations for treating ICI-mediated irAEs with steroids and secondary immunosuppressants.

1. Perform the Mantoux test (or an interferon gamma release assay [IGRA]) if it is not conducted at the baseline and there are no contraindications. Required prior to infliximab and vedolizumab administration Waiting for the test results should be balanced with the urgency of needing to treat the patient
2. Consider a proton-pump inhibitor (PPI) for gastrointestinal protection while on steroids (except in cases of suspected/biopsy-proven acute interstitial nephritis where PPIs should be avoided [18,19]; in those cases, consider H2-antagonists (e.g., famotidine)).
3. Prophylaxis against pneumocystis jirovecii pneumonia (PJP) can be considered. Trimethoprim/sulfamethoxazole * 1 double-strength tablet 3 × per week (or atovaquone if there is a sulpha allergy) if patients will be on prednisone (or equivalent) ≥ 20 mg for ≥1 month, especially if the patient has associated T cell defects, or is receiving other cytotoxic drugs or anti-TNF agents [20].
4. Perform a baseline HbA1C and glucose levels test to rule out undiagnosed diabetes; if it is abnormal, consider the following: Referring the patient to a family physician or endocrinologist for co-management. Prescribing glucometer and glucose monitoring strips, if needed. Providing education on glucose monitoring. Starting metformin if there are no contraindications.
5. Consider the Canadian guidelines for the management of osteoporosis and counsel on bone health and/or management if the prednisone (or equivalent) dose is > 5 mg daily for >3 months in the past year [21].
6. Maintain proactive weekly communication with the patient during the steroid taper until two weeks after the completion of steroids.

* Caution: Trimethoprim/sulfamethoxazole can induce Stevens–Johnson syndrome. Although dosing is included, clinicians should refer to the appropriate product monograph for dosing instructions.

**Table 2:** Management approach for ICI-mediated colitis.

Clinical Questions to Grade the Severity of Colitis			
Frequency of stools or output from ostomy? Presence of nocturnal bowel movements? Presence of blood in stool?			Presence of abdominal pain? Ability to carry out activities of daily living (ADL)?
Grade 1	Signs and Symptoms	Increase in stool frequency < 4/day over baseline Mild increase in ostomy output compared with baseline	
	Immediate Action	Inform the MD/RN care team.	
	Investigations	Ask about nocturnal bowel movements Order stool sample for C and S, C difficile, and ova and parasite	
	Management	Manage symptoms: ○ Prescribe loperamide if no contraindications ○ Counselling on dietary modifications and oral hydration Treatment with ICI can continue Ensure close monitoring	
Grade 2	Signs and Symptoms	Increase of 4–6 stools/day over baseline Moderate increase in output in ostomy compared with baseline Abdominal pain, blood or mucus in stool Nocturnal bowel movements Limiting self-care ADL	
	Immediate Action	Inform the MD/RN care team Hold ICI	
	Investigations	Ask about nocturnal bowel movements Order stool sample for C and S, C difficile, and ova and parasite Abdominal X-ray or, if abdominal pain and/or blood in stool, consider a CT of the abdomen/pelvis Send a referral to an endoscopist familiar with immune related colitis (specify on referral need for endoscopy and biopsy for confirmation of colitis) with a target referral time of <1–2 weeks ○ If the patient declines to have a confirmatory endoscopy or it is not appropriate to have an endoscopy, consider requesting a calprotectin stool test if applicable and available	
	Management	Manage symptoms: ○ Prescribe loperamide ○ Counselling on dietary modifications and oral hydration ▪ If symptoms were of a short interval with increased bowel movements and no nocturnal bowel movements or abdominal pain, consider 24 h of loperamide and then re-evaluation before proceeding with additional steps Start prednisone 1–2 mg/kg PO QD until bowel movements are back at the baseline, then slowly taper prednisone over 4 weeks. If not responsive to steroids in 48 h or refractory to the steroid taper and no contraindications, prescribe IV infliximab (5 mg/kg) or vedolizumab (300 mg) *	
Grade 3	Signs and Symptoms	Increase of ≥7 stools/day over baseline Nocturnal bowel movements Bowel incontinence Severe abdominal pain Significant increase in ostomy output compared with baseline Limiting self-care ADL	
	Immediate Action	Inform the MD/RN care team. Discontinue ICI Admit to hospital	
	Investigations	Ask about nocturnal bowel movements Order stool sample for C and S, C difficile, and ova and parasite Order CT abdomen/pelvis ○ Based on CT findings and if the patient is having abdominal pain, consider a consultation with a general surgeon, if required Consultation with a gastroenterologist for endoscopy and biopsy ○ If endoscopy is not conducted as an in-patient, send a referral for outpatient endoscopy with biopsy to confirm the diagnosis within a target referral time of <1–2 weeks. ○ If the patient declines to have a confirmatory endoscopy or it is not appropriate to have an endoscopy, consider requesting a calprotectin stool test if applicable and available	
	Management	Start IV hydration Consider IV antibiotics as per local institutional guidelines, and re-access once confirmation of immune related colitis. Start IV methylprednisone 2 mg/kg/daya. ○ Continue prednisone or methylprednisone 1–2 mg/kg/day until bowel movements return to the patient’s baseline, then slowly taper prednisone over 4 weeks. If not responsive to steroids in 48 h or refractory to the steroid taper and no contraindications, prescribe IV infliximab (5 mg/kg) or vedolizumab (300 mg) *	
Grade 4	Signs and Symptoms	Grade 3 plus fever or peritoneal signs that are consistent with bowel perforation or ileus	
	Immediate Action	Inform the MD/RN care team. Discontinue ICI Admit to hospital	
	Investigations	Order stool sample for C and S, C difficile and ova and parasite Order CT abdomen/pelvis ○ Based on CT findings and if the patient is having abdominal pain, consider a consultation with a general surgeon, if required Consultation with a gastroenterologist for endoscopy and biopsy ○ If endoscopy is not conducted as an in-patient, send a referral for outpatient endoscopy with biopsy to confirm the diagnosis within a target referral time < 1–2 weeks.	
	Management	Start IV hydration Start IV antibiotics as per local institutional guidelines, and re-access once confirmation of immune-related colitis Start IV methylprednisone 2 mg/kg/day ○ Continue prednisone or methylprednisone 1–2 mg/kg/day until bowel movements return to the patient’s baseline, then slowly taper prednisone over 4 weeks. If not responsive to steroids in 48 h or refractory to the steroid taper and no contraindications, prescribe IV infliximab (5 mg/kg) or vedolizumab (300 mg) *	

* If diarrhea or colitis symptoms persist after the second dose of infliximab, the third dose should be withheld, and three doses of vedolizumab (300 mg) should be administered at 0, 2, and 6 weeks [50].

**Table 3:** Management Approach for ICI-mediated Hepatitis.

Clinical Questions to Grade the Severity of Hepatitis		
Increase in alanine transaminase (ALT) and /or increase in bilirubin Increase in alkaline phosphatase (ALP) might indicate IR-cholangitis		
Grade 1	Signs and Symptoms	ALT: >ULN—3.0 × ULN and/or Bilirubin: >ULN—1.5 × ULN and/or ALP: >ULN—2.5 × ULN
	Immediate Action	Inform the MD/RN care team Close observation Discontinue hepatotoxic prescription/OTC/herbal medications, if possible
	Investigations	If hepatotoxic drugs were discontinued, consider repeating liver enzyme tests q3 days to determine if any improvement Order hepatitis B surface antigen, hepatitis B core antibody, and hepatitis C serology if not performed at baseline
	Management	Ensure close monitoring Treatment with ICI can continue ○ Consider holding ICI if ALT and/or bilirubin increases to borderline of grade 2 Repeat liver enzyme tests q3 days; continue to hold if ALT and/or bilirubin continue to increase
Grade 2	Signs and Symptoms	ALT: 3.0–5.0 × ULN and/or Bilirubin: 1.5–3.0 × ULN and/or ALP: 2.5–5.0 × ULN
	Immediate Action	Inform the MD/RN care team Hold ICI Discontinue hepatotoxic prescription/OTC/herbal medications, if possible
	Investigations	If hepatotoxic drugs were discontinued, consider repeating liver enzyme tests q3 days to determine if any improvement Order hepatitis B surface antigen, hepatitis B Core antibody, and hepatitis C serology if not performed at baseline Order liver ultrasound
	Management	Administer 0.5 to 1 mg/kg/d prednisone or equivalent until symptoms improve to grade ≤ 1 on steroid, then slowly taper steroids over 4–6 weeks ○ Monitor liver enzymes q3 days until the documented reduction in ALT, then continue to monitor weekly until off all corticosteroids ▪ Admission to a hospital if monitoring of liver enzymes as out-patient is not possible or concerned about compliance or the patient unwell and not deemed appropriate for out-patient management Start mycophenolate mofetil 500 mg to 1000 mg PO BID * only if: ○ Sustained elevation is significant, with two consecutive rises in liver enzymes and/or ○ Refractory to the dose of prednisone of 0.5–1 mg/kg/day without reduction in liver enzymes in 3 days or ○ During the steroid taper, liver enzymes start to rise with two consecutive tests Once symptoms and ALT/bilirubin improve to grade ≤ 1 on steroid, start slow steroid taper over 4–6 weeks. ○ Once prednisone is tapered off, decrease mycophenolate mofetil by 250–500 mg every week.
Grade 3	Signs and Symptoms	ALT: >5.0–20.0 × ULN and/or Bilirubin: >3.0–10.0 × ULN and/or ALP: >5.0–20 × ULN
	Immediate Action	Inform the MD/RN care team Discontinue ICI Discontinue hepatotoxic prescription/OTC/herbal medications, if possible
	Investigations	Order hepatitis B surface antigen, hepatitis B Core antibody, and hepatitis C serology if not performed at baseline Order liver ultrasound
	Management	Administer 1–2 mg/kg/d prednisone or equivalent until symptoms improve to grade ≤ 1 on steroid, then slowly taper steroids over 4–6 weeks ○ Monitor liver enzymes q3 days until the documented reduction in ALT, then continue to monitor weekly until off all corticosteroids ▪ Admission to a hospital if monitoring of liver enzymes as out-patient is not possible or concerned about compliance or the patient is unwell and not deemed appropriate for out-patient management Start mycophenolate mofetil 500 mg to 1000 mg PO BID * only if: ○ Sustained elevation is significant, with two consecutive rises in liver enzymes and/or ○ Refractory to the dose of prednisone of 1–2 mg/kg/day without reduction in liver enzymes in 3 days or ○ During the steroid taper, liver enzymes start to rise with two consecutive tests Once symptoms and ALT/bilirubin improve to grade ≤ 1 on steroid, start slow steroid taper over 4–6 weeks. ○ Once prednisone is tapered off, decrease mycophenolate mofetil by 250–500 mg every week. ○ Consider liver biopsy if liver enzymes do not improve/refractory to prednisone +/− mycophenolate mofetil.
Grade 4	Signs and Symptoms	ALT: >20.0 × ULN and/or Bilirubin: >10.0 × ULN and/or ALP: >20 × ULN
	Immediate Action	Inform the MD/RN care team Discontinue ICI Admit to hospital
	Investigations	Order hepatitis B surface antigen, hepatitis B Core antibody, and hepatitis C serology if not performed at baseline Order liver ultrasound
	Management	Administer 1–2 mg/kg/d prednisone or equivalent until symptoms improve to grade ≤ 1 on steroid, then slowly taper steroids over 4–6 weeks ○ Monitor liver enzymes q3 days until the documented reduction in ALT, then continue to monitor weekly until off all corticosteroids ▪ Admission to a hospital if monitoring of liver enzymes as out-patient is not possible or concerned about compliance or the patient is unwell and not deemed appropriate for out-patient management Start mycophenolate mofetil 500 mg to 1000 mg PO BID * only if: ○ Sustained elevation is significant, with two consecutive rises in liver enzymes and/or ○ Refractory to the dose of prednisone of 1–2 mg/kg/day without reduction in liver enzymes in 3 days or ○ During the steroid taper, liver enzymes start to rise with two consecutive tests Once symptoms and ALT/bilirubin improve to grade ≤ 1 on steroid, start slow steroid taper over 4–6 weeks. ○ Once prednisone is tapered off, decrease mycophenolate mofetil by 250–500 mg every week. ○ Consider liver biopsy if liver enzymes do not improve/refractory to prednisone +/− mycophenolate mofetil.

* Other immunosuppressants listed in the ESMO guidelines include tocilizumab 8 mg/kg, tacrolimus, azathioprine cyclosporine, or anti-thymocyte globulin (100 mg divided over 2 days).

**Table 4:** Management approach for ICI-mediated nephritis.

Clinical Questions to Grade the Severity of Nephritis		
Increase in creatinine or proteinuria?		
Grade 1	Signs and Symptoms	Creatinine increased > ULN—1.5 × ULN of the patient’s baseline Proteinuria 1+, <1.0 g/24 h
	Immediate Action	Inform the MD/RN care team Close observation Discontinue nephrotoxic drugs, PPIs/pantoprazole/OTC (NSAIDs)/herbal medications
	Investigations	Clinic exam Assess volume status and ask about urine output Order urinalysis and microscopy (if available) ○ Urine albumin/creatinine ratio, urine electrolytes (Na, K^+^, Cl, bicarbonate, Mg, Ca, PO_4_) ○ If the urinalysis is positive for protein (even 1+), order urine albumin–creatinine ratio/24 h urine protein collection Consider a trial of IV hydration (1 L of IV 0.9% normal saline) daily × 3 days Ultrasound of kidneys upfront or after trial of hydration if no improvement Repeat creatinine post hydration; if it rises to the level of grade ≥ 2, treat accordingly
	Management	Treatment with ICI can be continued Close monitoring of creatinine and urine protein by urinalysis (Q1–2 weekly) If creatinine and/or proteinuria increase to grade ≥ 2, treat accordingly
Grade 2	Signs and Symptoms	Creatinine > 1.5–3.0 × ULN of patients’ baseline Proteinuria 2+, 1.0–3.4 g/24 h
	Immediate Action	Inform the MD/RN care team Hold ICI * Discontinue nephrotoxic drugs, PPIs/pantoprazole/OTC (NSAIDs)/herbal medications
	Investigations	Clinic exam Assess volume status and ask about urine output Urinalysis ○ Electrolytes, extended biochemistry if not performed (Na, K+, Cl-, bicarbonate, Mg, PO4, calcium Consider serologies (ANA, ANCA) if other signs of systemic disease Order 24 h urine protein collection if urinalysis is positive for protein ○ Patients with proteinuria should be consulted by nephrology for direction on ACE/ARB Consider a trial of IV hydration (1 L of IV 0.9% normal saline) daily × 3 days depending on changes in the patient’s creatinine and proteinuria Repeat creatinine post hydration, and if persistent elevation or increase, continue with the escalation of management ○ Referral to nephrology if starting steroids Renal ultrasound Consult nephrology for consideration of biopsy
	Management	Start prednisone 1 mg/kg PO QD until creatinine is within normal limits or the patient’s baseline and proteinuria is normal or back to the patient’s baseline or grade 1; then slowly taper prednisone over 4 weeks ○ Monitor creatinine Q3 days until documented reduction then weekly until off corticosteroids ▪ Admission to the hospital if monitoring of creatinine as an out-patient is not possible or concerned about compliance or the patient is unwell and not deemed appropriate for out-patient management Add secondary immunosuppressant (standard option is mycophenolate mofetil, dose 500–1000 mg PO BID) ** only if: ○ Sustained significant creatinine elevation with two consecutive rises in creatinine levels and/or ○ Refractory to prednisone 1–2 mg/kg/day without reduction in creatinine in 1–2 weeks or ○ During the prednisone taper, creatinine or proteinuria start to rise with two consecutive tests (may see a rise in creatinine in the first week when starting secondary immunosuppressants) Biopsy prior to secondary immunosuppressant is strongly recommended as findings may inform choice of agent
Grade 3	Signs and Symptoms	Creatinine 3.0–6.0 × ULN of baseline Proteinuria ≥ 3.5 g/24 h
	Immediate Action	Inform the MD/RN care team Discontinue ICI Admit to hospital Referral to nephrology, consider kidney biopsy Discontinue all nephrotoxic prescriptions, including pantoprazole/OTC/herbal medications
	Investigations	Order urinalysis and microscopy (if available) Order 24 h urine protein collection if urinalysis is positive for protein
	Management	Start IV methylprednisone or prednisone 1–2 mg/kg/day ○ Continue until creatinine is within normal limits or the patient’s baseline and proteinuria is normal or back to the patient’s baseline or grade 1, then slowly taper over 4 weeks. ○ Consider 3 days of IV methylprednisone, then switching to oral equivalent if there are no concerns with oral absorption. Add secondary immunosuppressant (standard option is mycophenolate mofetil, dose 500–1000 mg PO BID) ** only if: ○ Sustained significant creatinine elevation with two consecutive rises in creatinine levels and/or ○ Refractory to prednisone 1–2 mg/kg/day without reduction in creatinine in 5 days or ○ During the prednisone taper, creatinine or proteinuria start to rise with two consecutive tests (may see a rise in creatinine in the first week when starting secondary immunosuppressants) Biopsy prior to secondary immunosuppressant is strongly recommended as findings may inform choice of agent If refractory to prednisone and mycophenolate mofetil, consider a third-line immunosuppressant+ such as infliximab, cyclophosphamide, tocilizumab or rituximab (if no contraindications), under the guidance of nephrology
Grade 4	Signs and Symptoms	Potentially life-threatening Creatinine > 6.0 × ULN
	Immediate Action	Inform the MD/RN care team Discontinue ICI Admit to hospital Urgent referral to nephrology as hemodialysis may be considered
	Investigations	Clinical exam for volume status and ask about urine output
	Management	Follow management of grade 3 nephritis

* Multicenter retrospective data suggest that approximately 20% of patients re-challenged with an ICI after ICI-related AKI may have recurrent AKI [31]. This suggests that many patients could potentially be considered for re-challenge, although the close monitoring of creatinine (every 1–2 weeks after restarting ICI) and cessation of PPIs/NSAIDs prior to re-challenge would be appropriate. ** Although infliximab has been suggested as preferred second-line option [80], more data are needed to consider infliximab over MMF or other second-line agents.

**Table 5:** Management approach for ICI-mediated myocarditis.

Clinical Questions to Grade the Severity of Myocarditis		
Symptoms and ability to carry out activities of daily living? Cardiac biomarkers? Electrocardiogram?		
Grade 1	Signs and Symptoms	Differentiate between an isolated troponin or ECG abnormality vs. asymptomatic LV dysfunction, as asymptomatic LV dysfunction is more specific (i.e., new LV dysfunction is less likely to be caused by other causes)
	Immediate Action	Inform the MD/RN care team Hold checkpoint inhibitor(s) Consider referral to a cardio-oncologist
	Investigations *	Troponin I B-type natriuretic peptide CRP CK ECG ^a^ Echocardiogram ^b^ Chest X-ray 72 h Holter monitor to look for malignant arrhythmias A cardiac MRI in cases where the diagnosis is not confirmed based on the above investigations.
	Management	No clear guidelines regarding immunosuppression Use clinical and biochemical features to determine if prednisone 1 mg/kg is required Restart checkpoint inhibitor(s) once grade 0
Grade 2–4	Signs and Symptoms	CTCAE v5.0: Ranging from symptoms with moderate activity or exertion to life-threatening consequences requiring urgent intervention. ASCO [11]: Ranging from mild symptoms with abnormal cardiac biomarkers and electrocardiogram to life-threatening consequences and either elevated cardiac biomarkers, depressed left ventricular ejection fraction or wall motion abnormalities, and/or diagnostic cardiac MRI
	Immediate Action	Inform the MD/RN care team Discontinue ICI Admit to the hospital and ensure the patient is on telemetry or has routine ECG monitoring if telemetry is not available. Refer to a cardio-oncologist for consideration of stress test, cardiac MRI, cardiac catheterization, and/or endomyocardial biopsy
	Investigations *	Troponin I B-type natriuretic peptide CRP CK ECG ^a^ Echocardiogram ^b^ Chest X-ray Cardiac MRI (if available) ○ Use the Lake Louise Criteria for using cardiac MRI to diagnose myocarditis [97,98,99]. Endomyocardial biopsy is not routinely recommended unless the diagnosis is unconfirmed
	Management	Ensure an adequate dose of steroids ○ Current guidelines, including ESMO as well as those developed by the European Society of Cardiology (ESC) [12,100], recommend high-dose pulse steroids such as IV methylprednisolone 0.5–1.0 g/day for 3–5 days as soon as possible, once the diagnosis of immune-related myocarditis is considered likely, to reduce the risk of major adverse cardiovascular events ** ▪ If clinical improvement is observed (troponin has fallen to <50% of peak level or to normal after 3 days of IV methylprednisolone) and the patient is clinically stable (no heart failure, ventricular arrhythmias, complete heart block), then conversion to oral prednisone 1 mg/kg/day), is recommended, reducing by 10 mg/week with troponin monitoring providing cardiovascular stability continues. ○ ASCO and CCO guidelines recommend high-dose corticosteroids (1–2 mg/kg of prednisone) initiated rapidly (oral or IV depending on symptoms) [11,14]. This alternative should be considered in consultation with multidisciplinary teams. ▪ Taper steroids for at least 4 to 6 weeks ▪ Consider ICI rechallenge if mild symptoms and complete recovery A multidisciplinary team discussion is recommended before restarting ICI treatment in patients with mild, clinically uncomplicated immune-related myocarditis If there is no improvement after 48–72 h of IV methylprednisolone, troponin is rising or <50% reduction from the peak or there is hemodynamic instability (CHF, cardiogenic shock, ventricular tachyarrhythmias) ○ Continue IV methylprednisolone 1 g/day ○ Add a second immunosuppressant, tocilizumab (8 mg/kg; one dose; every two weeks) [28], or MMF (500–1000 mg po/IV BID) [101] ○ Third-line options: anti–thymocyte globulin, alemtuzumab, or abatacept [102,103,104]. ** Monitoring ○ Monitor with ECG or telemetry (if still admitted to hospital), troponin +/− BNP, initially daily while admitted to hospital, then every 2–3 days, depending on clinical improvement. ○ Continue monitoring with troponin in an outpatient setting while tapering steroids and following completion of steroids +/− secondary immunosuppression for 1 month. ○ Review with/educate patient and family on symptoms to watch for

* Investigations: ^a^ ECG: look for the new prolongation of the PR interval, atrioventricular block, ventricular arrhythmias, frequent premature ventricular complexes, ST depression, or diffuse T-wave inversions increase in QRS prolongation [70]. ^b^ Echocardiogram: depressed left ventricular ejection fraction [89], regional wall motion abnormalities [96], diastolic dysfunction [96], and pericardial effusion [90]. ** Consult the advanced heart failure team for assistance (start on mycophenolate mofetil 500–1000 mg po/IV BID). Note: infliximab was not included as it has been associated with heart failure and is contraindicated at high doses in patients with moderate–severe heart failure [26].

**Table 6:** Endocrine evaluation and patient education.

**Timing**	**Tests**
Before the initial ICI administration	History, blood pressure, fluid intake, sodium, potassium, creatinine, AM cortisol, ACTH, TSH, free T4, prolactin, FSH, LH, total testosterone (males), estradiol (premenopausal females)
Before each subsequent administration *	History, blood pressure, fluid intake, sodium, potassium, creatinine, TSH, free T4, AM cortisol, ACTH Check for signs and symptoms of hypophysitis (headaches, visual disturbances, fatigue, polyuria, cold intolerance, weakness, nausea, vomiting, dizziness, hypotension)
**Patient Education**	
Provide education regarding stress doses of hydrocortisone in the event of infection, trauma or another medical event Patients on steroids should be encouraged to wear a medical alert bracelet	

* At least once every four weeks; ACTH, adrenocorticotropic hormone; FSH, follicle-stimulating hormone; LH, luteinizing hormone; TSH, thyroid-stimulating hormone.

**Table 7:** Management approach for ICI-mediated hypophysitis.

Clinical Questions to Grade the Severity of Hypophysitis		
Symptoms (headaches, vision changes, mood changes, dizziness, fatigue)? Ability to carry out activities of daily living?		
Grade 1	Signs and Symptoms	Asymptomatic or mild symptoms Clinical or diagnostic observation only (headache, fatigue)
	Immediate Action	Inform the MD/RN care team Consult endocrinology
	Investigations	Physical examination: vitals, including postural vitals Diagnostic workup: ACTH, AM cortisol, TSH, free T4, LH/FSH, prolactin, total testosterone (males), estradiol (pre-menopausal females), and electrolytes (Na^+^/K^+^) MRI with pituitary (sella turcica) protocol
	Management	No steroids are needed for immune suppression Hormone replacement depending on the affected hormonal axis * If there is no enlargement of the pituitary gland, ICI may be continued with close monitoring If the pituitary gland is enlarged, treat it as grade 2
Grade 2 (Moderate)	Signs and Symptoms	Moderate symptoms (mood changes, headaches, presyncope, fatigue) Limiting age-appropriate instrumental ADLs
	Immediate Action	Inform the MD/RN care team Hold ICI Consult endocrinology
	Investigations	Physical examination: vitals, including postural vitals Diagnostic workup: ACTH, AM cortisol, TSH, free T4, LH/FSH, prolactin, total testosterone (males), estradiol (pre-menopausal females), and electrolytes (Na^+^/K^+^) MRI with pituitary (sella turcica) protocol
	Management	Start prednisone 0.5–1 mg/kg/day orally until symptoms improve to baseline or grade 1, then slowly taper over 6 weeks ○ When prednisone at 10 mg PO QD, switch the patient to hydrocortisone 20 mg (morning), 10 mg (mid-afternoon) hormone replacement depending on the affected hormonal axis * Withhold ICI until resolution to grade 0–1. Upon improvement, treatment may be resumed after corticosteroid taper. ICI can be continued in the presence of hormone replacement as long as no symptoms are present.
Grade 3 (Severe)	Signs and Symptoms	Severe or medically significant but not immediately life-threatening symptoms Hospitalization or prolongation of existing hospitalization indicated Limiting self-care ADLs
	Immediate Action	Follow grade 4
	Investigations	Follow grade 4
	Management	Follow grade 4
Grade 4 (Potentially life- threatening)	Signs and Symptoms	Urgent intervention required (severe ataxia)
	Immediate Action	Inform the MD/RN care team Hold ICI Admit to the hospital Refer to endocrinology Ophthalmology consultation if optic chiasm is affected
	Investigations	Diagnostic workup: ACTH, AM cortisol, TSH, free T4, LH/FSH, prolactin, total testosterone (males), estradiol (pre-menopausal females), and electrolytes (Na^+^/K^+^) MRI with pituitary (sella turcica) protocol
	Management	Start 1 mg/kg/day methylprednisolone (or equivalent) daily until symptoms improve to baseline or grade 1. ○ Step down to prednisone 1 mg/kg/day when stable and slowly taper over 6 weeks. Hormone replacement depending on the affected hormonal axis * Steroids should start several days prior to any thyroid replacement to prevent adrenal crisis. Assess the risk of opportunistic infection based on the duration of steroid taper. If the patient is stable with hormone replacement, consider resuming an ICI following consultation with endocrinology and oncology.

* Most patients who experience ≥ grade 2 hypophysitis fail to recover pituitary function and might require lifelong hormone replacement therapy [123]. While the recovery of the thyroid and gonadal axes has been described in 24% and 58% of patients, respectively (hence, there is a need for re-evaluation at follow-up), adrenal axis damage appears permanent.

**Table 8:** Management approach for ICI-mediated pneumonitis.

Clinical Questions to Grade the Severity of Pneumonitis		
Symptoms (dyspnea, cough, chest pain, increasing oxygen requirements)? Ability to carry out activities of daily living?		
Grade 1	Signs and Symptoms	Asymptomatic Confined to one lobe of the lung or <25% of lung parenchyma Clinical or diagnostic observations only
	Immediate Action	Inform the MD/RN care team
	Investigations	Document pulse oximetry resting and with ambulation ○ Re-assess in 1–2 weeks Chest CT with contrast (if diagnosed via chest X-ray) ○ If diagnosed by CT chest, consider a baseline chest X-ray to be able to improve access to monitor radiographically Infectious workup if infectious symptoms
	Management	May continue ICI Ensure close monitoring Review with patient and family symptoms to watch for, and remember to assess symptoms at every visit
Grade 2 (Moderate)	Signs and Symptoms	Mild/moderate new symptoms: dyspnea, cough, chest pain Limiting instrumental ADLs Medical intervention indicated
	Immediate Action	Inform the MD/RN care team Hold ICI
	Investigations	Document pulse oximetry at rest and ambulation Consider nasal swab and/or sputum culture for respiratory pathogens High-resolution CT chest with contrast, or consider CT pulmonary angiogram if pulmonary embolism is on the differential Referral to respirologist for monitoring and consideration of bronchoscopy and BAL
	Management	Start antibiotics according to local practices if suspicious of infection (fever, elevated neutrophil count, CRP) If there is no evidence of infection and/or no improvement with antibiotics after 48 h, add in prednisone 1 mg/kg/day orally until symptoms improve to baseline or grade 1, then slowly taper over 6 weeks If there is no improvement after 48–72 h of oral prednisone, treat as grade 3/4 Monitor ○ Monitor pulse oximetry (re-assess the patient within 2–3 days) ○ Monitor with chest X-ray, initially frequently and early, every 2–3 days, then space out frequency depending on clinical improvement ○ If ICI is being reintroduced, this should be delayed until there is ongoing radiographic resolution while the daily dose of steroids equals ≤10 mg of oral prednisone per day
Grade 3/4 (Severe)	Signs and Symptoms	Severe symptoms Increasing oxygen requirements Limiting self-care ADLs New or worsening hypoxia Involvement of all lung lobes or >50% of lung parenchyma Life-threatening respiratory compromise Acute respiratory distress syndrome
	Immediate Action	Inform the MD/RN care team Discontinue ICI permanently Admit to hospital Documentation of goals of care
	Investigations	Order infectious workup as clinically indicated (nasal swab for viral pathogens, sputum culture, blood culture, and urine antigen test for legionella) Order a high-resolution chest CT scan or consider a CT pulmonary angiogram to rule out pulmonary embolism, if this remains on the differential Consult respirology for consideration of bronchoscopy and BAL If there remains diagnostic uncertainty, a lung biopsy can be considered with an understanding of the very high perioperative mortality associated with that procedure in this patient population [153].
	Management	Start IV antibiotics as per local institutional guidelines Start IV methylprednisone 2 mg/kg/day, or if severe hypoxia, consider pulse steroids with IV methylprednisone 1 g/day × 3–5 days ○ Continue IV methylprednisone 1–2 mg/kg/day (or start this dose after pulse steroids if given) until grade 1 or baseline, then convert to oral prednisone 1–2 mg/kg/day and taper over a minimum of 8 weeks ○ Repeat chest X-ray daily until clinically stable, and chest CT within 48–72 h of starting steroids If minimal to no improvement in 24–48 h of pulse steroids, add secondary immunosuppressants (Tocilizumab 4 mg/kg IV, preferred, can be repeated in 2–4 weeks) * if no contraindications + continue IV methylprednisone If minimal to no improvement with tocilizumab: Consider alternative immunosuppressants such as infliximab, mycophenolate mofetil, IVIG, or cyclophosphamide Monitor and discharge ○ Repeat chest CT with contrast in 3–4 weeks ○ At the time of discharge, refer to respirology with expertise in managing pneumonitis for ongoing monitoring post-discharge.

* Alternatives to tocilizumab include IVIG 2 g/kg over 2–5 days (preferred), infliximab 5 mg/kg × 2 doses (which can be repeated at 14 days), and/or cyclophosphamide 600 mg/m^2^ × 1 dose. For patients with steroid-dependent disease (i.e., an inability to taper steroids over 3–6 months), consider steroid-sparing agents such as mycophenolate mofetil 500–1500 mg PO BID.
